# Factors associated with the diagnosis of COVID-19 among Brazilian health professionals COVID-19 and health professionals

**DOI:** 10.1371/journal.pone.0267121

**Published:** 2022-06-24

**Authors:** Elucir Gir, Sheila Araújo Teles, Mayra Gonçalves Menegueti, Renata Karina Reis, Milton Jorge de Carvalho, Eliã Pinheiro Botelho, Laelson Rochelle Milanês Sousa, Hemilio Fernandes Campos Coelho, Fernanda Maria Pereira Ávila, Marli Teresinha Gimeniz Galvão, Wynne Pereira Nogueira, Ana Cristina de Oliveira e Silva

**Affiliations:** 1 Ribeirão Preto School of Nursing, University of São Paulo, Ribeirão Preto, São Paulo, Brazil; 2 College of Nursing, Federal University of Goiás, Goiânia, Goiás, Brazil; 3 Department of Gynecology and Obstetrics, College of Medicine of the ABC, Santo André, São Paulo, Brazil; 4 Federal University of Pará, Belém, Pará, Brazil; 5 Department of Statistics, Federal University of Paraíba, João Pessoa, Paraíba, Brazil; 6 Department of Nursing Rio das Ostras, Fluminense Federal University, Rio das Ostras, Rio de Janeiro, Brazil; 7 Department of Nursing, Federal University of Ceará, Fortaleza, Ceará, Brazil; 8 Postgraduate Program in Nursing, Federal University of Paraíba, João Pessoa, Paraíba, Brazil; 9 Department of Clinical Nursing, Federal University of Paraíba, João Pessoa, Paraíba, Brazil; University of New Mexico Health Sciences Center, UNITED STATES

## Abstract

**Introduction:**

Health professionals are on the front lines against the Coronavirus 2019 (COVID-19) pandemic and are at high risk for acquiring the infection. Failures in precautionary measures, inadequacy/scarcity of Personal Protective Equipment (PPE), and lack of social and family distancing may be associated with increased exposure and contamination by the new coronavirus. This study investigated the prevalence of COVID-19 among Brazilian health professionals and associated factors according to demographic and occupational characteristics.

**Methods:**

A cross-sectional, analytical study was conducted using an online survey with 12,086 health professionals from all regions of Brazil. Data were collected using an adaptation of the respondent-driven sampling method for the virtual environment. The outcome variable was the diagnosis of COVID-19. Bivariate and multiple logistic regression analyzes were used to identify an association between the diagnosis of COVID-19 and demographic and occupational variables. Variables were considered statistically significant based on p<0.05.

**Results:**

Most participants were female, from the northeast region, and nursing professionals. A prevalence of 31.95% (95%CI: 31.0%, 32.9%) of COVID-19 was estimated. Following multiple regression analysis, the variables associated with the diagnosis of COVID-19 among health professionals were: male gender, married individuals, professionals who provide care to patients with COVID-19, who work in a field hospital, and those who work in institutions that did not offer enough quality PPE.

**Conclusions:**

The study found a high prevalence of COVID-19 infection, with male professionals being those with greater chances. Inadequate supply or poor quality of PPE offered by health institutions compromises the health of professionals with an increase in positive diagnosis for COVID-19.

## Introduction

Severe acute respiratory syndrome Coronavirus 2 (SARS-CoV-2), responsible for the Coronavirus Disease pandemic of 2019 (COVID-19), has already affected 178,202,610 people worldwide and was responsible for 3,865,738 deaths [1]. Brazil currently represents the epicenter of the COVID-19 pandemic in Latin America.

The first case of the disease in Brazil was reported on February 26, 2020, in the state of São Paulo, in the country’s southeastern region, and soon spread uncontrollably to all states [2]. SARS-CoV-2 is mainly transmitted by respiratory via, although indirect transmission through contact with contaminated surfaces can occur [3]. Therefore, social distancing, masks, and hand hygiene are essential non-pharmacological strategies to mitigate the risk of viral transmission in the general population.

Health professionals, who are on the front lines against COVID-19, represent a subgroup of the population at high risk of acquiring new coronavirus infection at all levels of health care. For example, it is estimated that in China, more than 3,000 health professionals were infected with SARS-CoV-2, and 23 died from complications of the disease. In Italy, 4,824 professionals were diagnosed with COVID-19 with 24 physician deaths [4]. Furthermore, a meta-analysis showed an estimated prevalence for COVID-19 of 11% (95%CI: 7%, 15%) among 75,859 health professionals screened in Europe, the United States, and Asia [5].

The Americas has the most significant number of infected health workers. For example, in Brazil, 39,510 cases of COVID-19 were confirmed among health professionals. Among them, nursing technicians/auxiliaries (11,779), nurses (6,747), and physicians (4,690) were the most affected, followed by community health workers (1,941) and pharmacists (1845) [6].

A study suggests that SARS-CoV-2 infection in health professionals is associated with inadequate or failed precautionary/protective measures against the epidemic, lack of personal protective equipment (surgical masks, N95 type face masks, and protective gowns), working in crowded spaces, absence of social and family distance, infected and asymptomatic individuals having contact with health professionals, and other factors [7].

In Brazil, where the pandemic is still active, and vaccination against SARS-CoV-2 has been a slow process, researching the epidemiological situation of health professionals in the COVID-19 scenario is essential for implementing actions that mitigate the impact of the pandemic in a critical population to face this global health crisis. Therefore, this study aimed to investigate the prevalence of the diagnosis of COVID-19 among Brazilian health professionals and the associated factors according to demographic and occupational characteristics.

## Materials and methods

### Study design and period

A cross-sectional, analytical study was performed using an online survey, from October 1 to December 31, 2020, in all regions of Brazil. The study followed the recommendations of the do Strengthening the Reporting of Observational Studies in Epidemiology for RDS Studies (STROBE-RDS) [8] and was guided by the Checklist for Reporting Results of Internet E-Surveys (CHERRIES) [9].

### Study participants

Health professionals who worked in direct individual patient care situations, with or without the presence of COVID-19, in different health care scenarios in public or private services, at least in the last six months prior to the start of data collection, were considered eligible.

### Determining the sample size

The number of professionals per region of Brazil was the reference used to calculate the sample, with data from the professional councils provided by the Ministry of Health, base year 2010 [10]. A confidence interval of 95% was used, with a margin of error equal to +/- 1%, obtaining a minimum sample of 5,079 individuals. Following online data collection, 12,086 participants participated in the study. The calculation formula [11] is as follows:

$$n=\frac{1}{\left(\frac{1}{n_{0}}+\frac{1}{N}\right)},$$

in which

$$n_{0}=\frac{z^{2}S^{2}}{d^{2}}$$

In the formula presented:

- *z* is the value related to the confidence level established for the data collection (z = 1.96 for 95% confidence);
  - *N* is the size of the population;
  - *S* is the standard deviation;
  - *d* is the margin of error (pre-established according to the mean score to be calculated).

### Sampling procedure

Professionals were recruited using the Respondent Driven Sampling (RDS) method adapted for online environments. The participant is encouraged to recruit other individuals of the same professional category through social networks using this method. For this study, the data collection was performed by researchers selected from all regions of Brazil. These researchers went through four hours of pre-training to conduct an online survey during COVID-19.

This sampling method, developed and improved for HIV prevention studies [12–14], is based on Snowball Sampling and uses a mathematical model that weights the individuals in the sample according to their degree of social relationships to reduce selection bias and obtain reliable estimates in studies that involve obscured or difficult-to-reach populations. In this method, the participant is responsible for recruiting other individuals.

The RDS method used in this study was performed as follows: a random selection of a set of participants was made (seeds). The seeds were limited to ten referrals each in the first selection, and they managed that data in an Excel spreadsheet. Referral slots were limited to leave enough candidates in the pool to continue the referral chain as long as possible within their networks. Once the seeds made their referrals, each participant who returned the contact via WhatsApp was interviewed and received similar training to manage the spreadsheet with the ten indications to be made. A new selection round was generated when the referral spreadsheets were returned to the seeds. This back-and-forth took the place of physical coupons and allowed the data collection to be managed remotely, which was necessary due to the COVID situation.

It is essential to clarify that, in the way it was planned, the sampling plan did not consider the national region since the researchers did not control the onward recommendations. Therefore, this variable is included as a domain of interest and not a genuine stratum with a sample planned for that purpose.

### Data collection techniques and tools

A pilot test was conducted where individuals were contacted through social media applications and asked to participate in the pilot study. Subsequently, 47 respondents were invited to send feedback or comments on the survey via WhatsApp. All suggested changes were considered, in addition to minor adaptations to the terminology.

After the pilot study, data collection began, and a link was sent to access the Free and Informed Consent Form (FICF) and the data collection form (created and validated—form and content, by fifteen experts). The completed instruments were hosted on the SurveyMonkey platform, which allowed one single submission of the form via IP (Internet Protocol), enhancing the security of the collected information.

The instrument included multiple-choice questions, some of which were mandatory to proceed, divided according to demographic variables, as well as variables related to the professional category, the type of care provided, variables related to the availability, quality, and access of PPE, and variables related to the use of masks and preventive measures in the home environment.

### Study variables

The outcome variable was the diagnosis of COVID-19 according to a laboratory diagnostic report of detection of RNA-SARS-CoV-2 or anti-SARS-CoV-2 antibodies. The independent variables were sex, age, profession, family life, working in a field hospital, whether patient care is provided with or without COVID-19, access to and quality of PPE, reuse of surgical and N-95 masks.

### Data analysis and processing

The collected data were exported and analyzed using the R statistical software, version 4.0.4. Descriptive analysis was performed using frequency distribution and standard deviation. Prevalences were calculated with a 95% confidence interval (95%CI). The Chi-square test was used to test differences between proportions. Statistically significant associations were considered with values of p < 0.05. A logistic regression model was initially adjusted [15], and from this adjustment, the variables with a p-value < 0.20 were included in a new adjustment by the stepwise method to estimate the odds ratio (OR). The model presented in this work is the final model after applying the method. The significance level adopted was 5%. The variance inflation factor test was used [16] to check multicollinearity. Finally, explanatory variables were tested for associations between them. In general, the results did not show statistical evidence of loss of quality of the variables explaining the adjustment, nor changes in interpretation regarding the value of the estimates of the model’s parameters. That is, there was evidence of the absence of multicollinearity.

### Ethical consideration

The project was approved by the Research Ethics Committee (CEP) under opinion n° 4,258,366. All ethical aspects were addressed according to Resolutions 466/2012 and 510/2016.

## Results

The study included 12,086 health professionals from all regions of Brazil, with a mean age of 35.14 years (SD = 9.39), (95%CI: 34.9%, 35.3%). A prevalence of COVID-19 was estimated at 31.95% (95% CI: 31.0%, 32.9%). That is, for every 100 health professionals, approximately 32 reported a positive diagnosis according to a laboratory diagnosis of RNA-SARS-CoV-2 detection or anti-SARS-CoV2 antibodies. Data are presented in Table 1.

**Table 1:** Sociodemographic characteristics of Brazilian health professionals according to COVID-19 diagnosis, Brazil, 2020 (N = 12,086).

Variables	COVID-19 Diagnosis	
	No (n = 8,222)	Yes (n = 3,864)
	n (%)	n (%)
**Region**		
North	956 (11.6)	822 (21.3)
North East	2,363 (28.7)	1,275 (33.0)
Midwest	1,402 (17.0)	726 (18.8)
Southeast	2,662 (32.4)	831 (21.5)
South	839 (10.3)	210 (5.4)
**Professional category**		
Doctor	935 (11.4)	363 (9.4)
Nurse	3,948 (48.0)	1,942 (50.,3)
Nursing professionals	2,035 (24.8)	1,114 (28.8)
Physiotherapist	469 (5.6)	201 (5.2)
Psychologist	156 (1.9)	31 (0.8)
Speech therapist	49 (0.6)	10 (0.3)
Occupational Therapist	30 (0.4)	9 (0.2)
Dentist	192 (2.3)	48 (1.2)
Other	408 (5.0)	146 (3.8)
**Ethnicity**		
White	4,263 (51.8)	1,672 (43.3)
Black	632 (7.7)	323 (8.4)
Brown	3,197 (38.9)	1,807 (46.8)
Asian	130 (1.6)	62 (1.5)
**Religion**		
Have religion	7,179 (87.3)	3,396 (87.9)
No religion	1,043 (12.7)	468 (12.1)

Most professionals in the study adopted preventive measures in the home environment. Among these, the most frequent were food hygiene, 8,606 (71.2%), and fabric masks, 11,513 (95.26%). Prevention measures against COVID-19 used in the home environment were hand hygiene, 11,184 (92.5%), followed by cleaning, 6,364 (52.7%). It is noteworthy that other measures were listed less frequently, with the use of N-95 masks, 1,685 (13.9%), physical distance from family members 5,283 (43.7%), separation of household utensils, 2,124 (17.6%), and home isolation 2,092 (17.3%). It is noteworthy that 544 (4.5%) professionals changed their homes to prevent COVID-19 in the home environment.

Regarding personal protective equipment related to the reuse of N-95 masks, of the 12,086 participants, 2,094 (17.3%) professionals reported changing them from 0 to five days, 2,939 (24.3%) from six to 10 days, 3,511 (29.1%) from 11 to 15 days, 138 (1.1%) from 16 to 20 days, 23 (0.2%) from 20 to 25 days, 976 (8.1%) from 26 to 30 days, 56 (0.5%) over 30 days, and 2,349 (19.4%) did not report the frequency of exchange. Regarding the time to change surgical masks, 2,183 (18.1%) professionals reported changing the mask at the end of their shift, 309 (2.6%) changed it every hour, 2,048 (16.9%) every two hours, 1,069 (8.8%) every three hours, 2,242 (18.6%) every four hours, 1,837 (15.2%) reported changing at another frequency, and 2,398 (19.8%) reported not using such a mask.

When analyzing the factors associated with having a diagnosis of COVID-19, Table 2 presents the results found.

**Table 2:** Bivariate analysis of potential factors associated with the diagnosis of COVID-19 among health professionals, Brazil, 2020 (N = 12,086).

Variables	COVID-19 Diagnosis		p-value*
	No (n = 8,222) n (%)	Yes (n = 3,864) n (%)	
**Sex**			**< 0.001**
Male	1,531 (64.0)	860 (36.0)	
Female	6,691 (69.0)	3,004 (31.0)	
**Marital Status**			**0.10**
Single/Divorced	3,968 (69.0)	1,786 (31.0)	
Married/Stable union	4,204 (67.2)	2,056 (32.8)	
Widower	50 (69.4)	22 (30.6)	
**Patients served**			**< 0.001**
Patients with COVID-19	1,276 (63.6)	730 (36.4)	
Patients without COVID-19	3,382 (71.0)	1,381 (29.0)	
Both	3,564 (67.0)	1,753 (33.0)	
**Work in a field hospital**			**< 0.001**
Yes	2,198 (61.5)	1,376 (38.5)	
No	6,024 (70.8)	2,488 (29.2)	
**Adopted protective measures in family life**			**< 0.001**
Yes	7,837 (68.1)	3,676 (31.9)	
No	194 (61.8)	120 (38.2)	
Not applicable	191 (73.7)	68 (26.3)	
**Sufficient PPE provided by the workplace**			**< 0.001**
Yes	6,043 (69.4)	2,668 (30.6)	
No	403 (60.5)	263 (39.5)	
Somewhat	1,776 (65.6)	933 (34.4)	
**Good quality PPE provided by the workplace**			**< 0.001**
Yes	4,570 (69.8)	1,975 (30.2)	
No	818 (63.0)	481 (37.0)	
Somewhat	2,834 (66.8)	1,408 (33.2)	

*Teste Chi-square

In the association between the variables, the diagnosis of COVID-19 among health professionals was associated with gender (p < 0.001), with men having more COVID-19 than women. In addition, professionals who provide care to patients with COVID-19 had the diagnosis more frequently than those who provide care to patients in general (p < 0.001) and compared to those who provide care to both patients in general and patients with COVID-19. Professionals who worked in field hospitals also had the disease more frequently compared to those who did not work in one (p < 0.001).

In addition, professionals who adopted protective measures in their family life had COVID-19 less frequently than those who did not adopt such measures (p < 0.001). Furthermore, when the PPE provided by the institution was sufficient, health professionals had less frequency of COVID-19 compared to those in which the institution did not provide adequate PPE (p < 0.001) or provided only some. Finally, when professionals reported that the PPE offered was of good quality, they had the disease less frequently than those who reported that it was not of good quality (p < 0.001).

Table 3 shows the logistic regression model performed to assess the variables associated with the professional presenting the diagnosis of COVID-19.

**Table 3:** Odds ratios by logistic regression for the diagnosis of COVID-19 among health professionals, Brazil, 2020.

Variables	Crude OR (CI = 95%)	p-value	Adjusted OR (CI = 95%)	p-value
Male	1.25 (1.14–1.37)	< 0,001	**1.23 (1.12–1.36)** *	**< 0.001**
People who care for patients with COVID-19 compared to the general public	1.40 (1.25–1.56)	< 0.001	**1.27 (1.13–1.43)** *	**< 0.001**
People who provide assistance to both (with and without COVID-19) compared to those who provide service to the general public	1.20 (1.11–1.31)	< 0.001	**1.15 (1.06–1.26)** *	**< 0.001**
Field Hospital Service (yes)	1.52 (1.40–1.64)	< 0.001	**1.49 (1.37–1.62)** *	**< 0.001**
Preventive measures in family life (no)	1.32 (1.05–1.66)	0.020	**1.29 (1.,02–1.63)** **	**0,030**
Provision of sufficient PPE by the workplace (no)	1.48 (1.26–1.74)	< 0.001	**1.40 (1.15–1.78)** *	**< 0.001**
Provision of sufficient PPE by the workplace (in part)	1.18 (1.08–1.29)	< 0.001	**1.18 (1.05–1.37)** *	**< 0.001**
Provision of good quality PPE by workplace (no)	1.36 (1.20–1.54)	< 0.01	**1.20 (1.03–1.40)** **	**0.002**

*p-value < 0.001

**p-value < 0.05

Crude OR = Crude Odds Ratio; OR adjusted = Odds Ratio adjusted

The chances of males having a positive diagnosis for COVID-19 increased by 1.23 times compared to females. Regarding the type of patient, the chances of receiving a positive diagnosis increased by 1.27 and 1.15 times, respectively, for professionals who exclusively care for patients with COVID-19 compared to those who care for the general public and those who care for both profiles of patients with and without a diagnosis of COVID-19. Having provided care in a field hospital increases the chance of receiving a positive diagnosis for COVID-19 by 1.49 times.

Not adopting preventive measures in family life increases the chance of having a positive diagnosis for COVID-19 by 1.29 times compared to professionals who adopt preventive measures in family life. Insufficient PPE provided in the workplace increases the chance of receiving a positive diagnosis for COVID-19 by 1.40 times compared to institutions that provided sufficient PPE. Furthermore, the results show that the institution that has provided "some" sufficient PPE increases the chance of receiving a positive diagnosis for COVID-19 by 1.18 times compared to institutions that provided sufficient PPE. Regarding quality, not providing good quality PPE increases the chance of receiving a positive diagnosis for COVID-19 by 1.20 times compared to institutions that provided good quality PPE.

Table 4 describes the logistic regression performed to assess the variables associated with the health professional presenting a diagnosis of COVID-19 by region of Brazil.

**Table 4:** Odds ratios by logistic regression for the diagnosis of COVID-19 according to regions of Brazil, 2020.

Variables	Crude OR (CI = 95%)	p-value	ORadj (CI = 95%)	p-value
**Northeast region**				
Male	1.26 (1.06–1.49)	0.01	**1.22 (1.03–1.46)** *	**0.02**
Marital status (married)	1.11 (0.97–1.28)	0.12	**1.16 (1.01–1.33)** *	**0.03**
Field Hospital Service (yes)	1.46 (1.27–1.68)	< 0.001	**1.46 (1.26–1.70)** **	**< 0.001**
**North region**				
People who care for patients with COVID-19 compared to the general public	1.65 (1.25–2.17)	< 0.001	**1.54 (1.16–2.06)** **	**< 0.001**
Field Hospital Service (yes)	1.49(1.23–1.82)	< 0.001	**1.43 (1.17–1.76)** **	**< 0.001**
Preventive measures in family life (no)	2.08 (1.05–4.13)	0.03	**2.21 (1.10–4.43)** *	**0.03**
Sufficient PPE provided by the workplace (no)	1.38 (0.99–1.91)	0.05	**1.57 (1.06–2.33)** *	**0.02**
Sufficient PPE provided by the workplace (some)	1.28 (1.04–1.58)	0.02	**1.33 (1.03–1.73)**	**0.03**
**Midwest region**				
Male	1.33 (1.08–1.66)	< 0.001	**1.33 (1.07–1.65)** *	**0.01**
Field Hospital Service (yes)	1.44 (1.19–1.75)	< 0.001	**1.38 (1.13–1.69)** **	**< 0.001**
Good quality PPE provided by the workplace (some)	1.08 (0.87–1.35)	0,46	**1.30 (1.04–1.63)** *	**0.02**
**Southeast region**				
Marital status (married)	1.29 (1.10–1.51)	< 0.001	**1.32 (1.12–1.55)** **	**< 0.001**
People who care for patients with COVID-19 compared to those who care for both (with and without COVID-19)	1.36 (1.08–1.73)	< 0.001	**1.31 (1.02–1.68)** **	**< 0.001**
People who care for both (with and without COVID-19) compared to those who care for patients without COVID-19	1.40 (1.18–1.66)	< 0.001	**1.41 (1.19–1.68)** **	**< 0.001**
Field Hospital Service (yes)	1.28 (1.08–1.53)	< 0.001	**1.24 (1.03–1.50)** **	**< 0.001**
Good quality PPE provided by the workplace (no)	1.60 (1.25–2.04)	< 0.001	**1.38 (1.02–1.87)** *	**0.03**
**South region**				
Field Hospital Service (yes)	1.67 (1.20–2.31)	< 0.001	**1.65 (1.17–2.32)** **	**< 0.001**
Sufficient PPE provided by the workplace (no)	3.53 (1.55–8.02)	< 0.001	**3.63 (1.38–9.51)** *	**0.01**

*p-value < 0.05

**p-value < 0.001, Crude OR = Crude Odds Ratio; ORadj = Adjusted Odds Ratio

In all regions, a health professional who worked in field hospitals to treat COVID-19 increased their chances of receiving a diagnosis for COVID-19

As for PPE, the results show that in the North, Southeast, Midwest, and South, quality PPE or insufficient quantity increased the chances for diagnosing COVID-19. However, for the Northeast region, these variables referring to the quantity or quality of PPE did not show a statistically significant association.

On the other hand, the marital status variable showed a statistically significant association only for professionals in the Northeast region. Being married increases the chances of being diagnosed with the infection by 1.16 times for professionals in this region.

## Discussion

This study identified the prevalence of COVID-19 among Brazilian health professionals and associated factors according to demographic and occupational characteristics. The factors associated with the diagnosis of COVID-19 among health professionals were male gender, professionals who provide care to patients with COVID-19, who work in a field hospital, and those who work in institutions that offer insufficient PPE. These findings were also observed in another Brazilian study conducted in the state of São Paulo [17].

Health professionals have been involved since the beginning, in the emergency care and control of COVID-19, at all levels of health care, public or private, working hard and tirelessly to guarantee care 24 hours a day. As a result, even with preventive measures in the workplace, COVID-19 is increasingly prevalent in this professional category.

In this context, the prevalence of COVID-19 among respondents in this survey was relatively high (31.95%), which is similar to another study carried out in Nicaragua (30.35%) [18]. However, in other European countries, the infection rate was lower [19, 20], mainly attributed to strict compliance with non-pharmacological measures such as hand hygiene and the use of PPE [21]. Studies show that the prevalence of COVID-19 among healthcare professionals can range from 8% to 31% [22, 23]. With vaccination, cases of COVID-19 have also decreased in health professionals. An investigation carried out in India found that in fully vaccinated health professionals the rate was close to 19% [24].

The most affected professional categories were nursing professionals, physicians, and physiotherapists. There was also a higher frequency of COVID-19 among women since most nurses and nursing professionals are women. However, male health professionals had greater chances of being diagnosed with COVID-19. This data corroborates a study that showed that men have a slightly higher risk than women, and professionals working in public services have a higher risk of prevalence of COVID-19 [25].

Prolonged exposure to many infected patients directly increases the risk of infection for health professionals, and in public services, patients are more vulnerable, a factor that enhances transmission [19, 22]. In addition, there is still pressure for treatment, and this intense and heavy work, in addition to the lack of rest, can indirectly increase the possibility of transmission of the infection to health professionals [25].

Current guidelines and protocols for the protection of health professionals should be strictly followed. The focus of protection must be based on carrying out specific training and encouraging adherence to precautionary measures, emphasizing the use of PPE, hand hygiene, disinfection of patient care areas, and waste management [26].

In Scotland, a survey conducted with 158,445 health professionals found that professionals who work on the front lines during the pandemic constitute, together with their families, a significant sample among cases admitted with a diagnosis of COVID-19 at the hospital level [27].

Healthcare professionals can be exposed to SARS-CoV-2, both in and out of the workplace, increasing the risk of infection. Among the main prevention measures adopted in family life, hand hygiene, cleanliness of the environment, food hygiene, fabric masks, distance, and isolation at home stood out. These measures are efficient non-pharmacological interventions to prevent the spread of the virus, emphasizing hand hygiene, the use of masks, and social isolation.

In this study, professionals who reported that the institution did not provide enough PPE had a higher risk of having COVID-19. Providing adequate and sufficient PPE is an essential strategy for reducing the risk of infection among health professionals. An integrative literature review highlighted that the lack of PPE and the lack of adequate training for its correct and consistent use were risk factors for the contamination of nursing professionals by the coronavirus [28]. In addition, lack of PPE and lack of training are risk factors for the high number of SARS-CoV-2 infections among healthcare professionals [29–31].

From this perspective, these results indicate how necessary it is to manage care for human life, whether the patient or the professional, whether in hospitals, clinics, or the family environment, especially that performed by the multidisciplinary team, such as nursing, medicine, physiotherapy, among others. Furthermore, performing these services highlights the need for government attention to the management of health care workers and professionals, realizing the inadequate conditions they live in to confront the pandemic [32].

### Limitations

The main limitation of this study is related to the recruitment of participants. As the research was performed online, there may have been an over-representation of professionals who are skilled in the use of computers and social networks. However, this did not interfere with the results, as the number of participants exceeded the minimum necessary.

## Conclusions

There was significant participation of 12,086 health professionals from different regions of Brazil, with a diagnosis of COVID-19 prevalence of 31.5%, with male professionals being more likely to be infected (p < 0.001).

The evidence reveals that in all regions of Brazil, health professionals who worked in a field hospital were more likely to have COVID-19. In addition, it has also been shown that not taking adequate preventive measures in family life increases the chances of a positive diagnosis for COVID-19 among family members.

Inadequate supply or poor quality of PPE offered by health care facilities compromises the health of professionals with an increase in positive diagnoses for COVID-19.

The results demonstrated inadequacies in the use of masks in different regions of Brazil, and these illnesses that occurred among professionals as a result of care for people with COVID-19 point to the need for a quality health system, with sufficient supplies for the prevention of COVID-19, in order to protect professionals and preserve the health workforce against this infection impacting the Brazilian health system.

## Supporting information

S1 Questionnaire (DOCX)

S2 Questionnaire (DOCX)
