# Supplementary material for: Factors associated with the diagnosis of COVID-19 among Brazilian health professionals COVID-19 and health professionals
Source: PLoS One. 2022 Jun 24;17(6):e0267121. doi: 10.1371/journal.pone.0267121 (PMC9231739; doi:10.1371/journal.pone.0267121)
Supplement: S1 Questionnaire — (DOCX) [file pone.0267121.s001.docx]

This document is not the same survey provided to research participants in the present study. It has been has been translated from Portuguese to English for the convenience of English language users. It has not been validated for use in the English language. Data input fields and links have been stripped and the document was formatted for display in text only.

**You are being invited to participate in a study to analyze physical, psychological and social effects resulting from the COVID-19 pandemic. You will not be identified at any time. We ask for your valuable contribution in answering the following items. The average expected time to respond is 10 minutes. Thank you!**

**Terms of free and informed consent:**

https://www.dropbox.com/s/7um6z3vv0mcqd1l/TCLE.pdf?dl=0

1. Do you agree to participate in this study ?

Yes

No

2. Are you a health care professional?

Yes

No. Thank you for your participation.

3. Do you work in direct patient care?

Yes

No. Thank you for your participation.

4. What types of patients do you treat?

With COVID (suspected or confirmed)

In general

Both

5. Professional category

Doctor

Nurse

Nurse Technician

Auxiliar de Enfermagem

Physiotherapy

Psychology

Speech therapy

Occupational Health

Odontology

Other

6. State

7. City

8. Sex

Male

Female

9. Skin color

Non-hispanic white

Non-hispanic black

Brown

Yellow

Red

10. Civil status

Single

Married

Divorced/Separated

Stable union/Living together

Widow(er)

11. Education

High school

University

Postgraduate

12. Religion

Catholic

Evangelical

Spiritist

None

Other

13. Specialty

Infectology

General Clinic

Obstetrics and gynecology

Surgery

Therapy

Psychology

Immediate care or emergency

Not applicable

Other

14. Area of practice

Outpatient

Intensive Care Unit

Nursing

Surgical Center

First Aid Center

Emergency Department

General clinic

Private office

Field hospital for patients with COVID-19

Other

15. What type of service do you work in?

Private

Public

Philanthropic

16. Have you ever worked in a COVID-19 field hospital

Yes

No

17. Did your workplace provide sufficient PPE?

Yes

No

Somewhat

18. Did your workplace provide high quality PPE?

Yes

No

Somewhat

19. Did you adopt COVID-19 preventive measures in your home and personal life?

Yes

No

Not applicable
