# Supplementary material for: Factors associated with the diagnosis of COVID-19 among Brazilian health professionals COVID-19 and health professionals
Source: PLoS One. 2022 Jun 24;17(6):e0267121. doi: 10.1371/journal.pone.0267121 (PMC9231739; doi:10.1371/journal.pone.0267121)
Supplement: S2 Questionnaire — (DOCX) [file pone.0267121.s002.docx]

# Você está sendo convidado para participar deste estudo que tem como objetivo analisar os efeitos físicos, psicológicos e sociais decorrentes da pandemia da COVID-19. Você não será identificado em nenhum momento. Solicitamos a sua valiosa colaboração em responder os itens que seguem. O tempo médio previsto para responder é 10 minutos. Muito obrigada!

**Termo de Consentimento Livre e**

**Esclarecido:** [**https://www.dropbox.com/s/7um6z3vv0mcqd1l/TCLE.pdf?dl=0**](https://www.dropbox.com/s/7um6z3vv0mcqd1l/TCLE.pdf?dl=0)

## 1. Você concorda em participar da pesquisa?


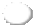

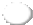
 Sim Não

## 2. Você é profissional da saúde?


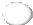
 Sim


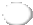
 Não. Agradecemos sua participação

## 3. Você atua na assistência?


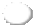
 Sim


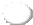
 Não. Agradecemos sua participação

## 4. Para que tipos de pessoas você presta assistência?


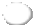

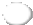
 Com COVID (suspeita ou confirmado)
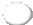
 Em geral

Ambos

## 5. Categoria profissional


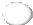
 Medico
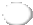
 Enfermeiro


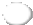
 Técnico de Enfermagem
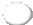
 Auxiliar de Enfermagem
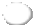
 Fisioterapeuta


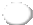
 Psicólogo


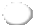
 Fonoaudiologo


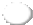
 Terapeuta ocupacional
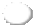
 Odontólogo


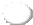
 Outro

##
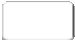
6. Idade

-
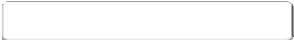
7. Estado
- 8. Cidade
  - 9. Sexo


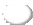
 Masculino
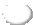
 Feminino

## 10. Cor da pele


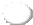

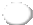
 Branca
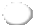
 Preta
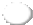
 Parda
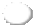
 Amarela

Vermelha

## 11. Estado conjugal


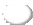
 Solteiro
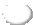
 Casado


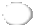
 Divorciado/separado


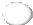
 União estável/morando junto
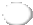
 Viúvo

## 12. Religião


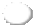
 Católico
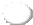
 Evangélico
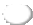
 Espírita
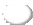
 Não tem
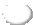
 Outra

## 13. Praticante


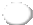
 Sim
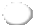
 Não

## 14. Escolaridade


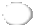
 Ensino Médio
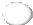
 Superior


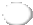
 Pós-graduação

## 15. Especialidade que atua


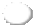
 Infectologia
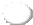
 Clínico geral


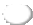
 Ginecologia e obstetrícia
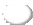
 Cirurgia


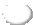
 Terapia intensiva
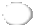
 Psiquiatria


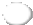

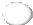
 Urgência e Emergência
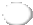
 Não se aplica

Outra

## Caso tenha respondido a opção "outra", especificar

1. Setor (es) de atuação

Ambulatório

Unidade de Terapia Intensiva Enfermaria

Centro cirúrgico

Unidade de Pronto Atendimento Unidade de Emergência Unidade Básica de Saúde Consultório particular

Hospital de campanha para assistência a pacientes com COVID-19 Outro

## 18. Tipo de instituição onde trabalha

Privada Pública Filantrópica

## 19. Você prestou assistência em hospital de campanha para a COVID-19?


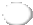
 Sim
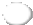
 Não

##
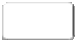
20. Tempo de experiência na função (em anos).

- -
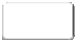
21. Tempo de formado (em anos).
  - 22. Você tem filhos?


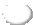

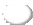
 Sim Não

## 23. Teve que se afastar dos filhos, familia para exercer a profissão?


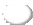
 Sim
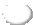
 Não


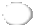
 Não se aplica

##
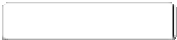
24. Quantas pessoas residem na mesma casa (incluindo você)?

- - 25. Há crianças menores de 12 anos que moram na mesma casa com você?


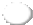
 Sim
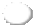
 Não

## 26. Há idosos ou pessoas em grupo de risco para COVID-19 que moram com você?


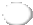
 Sim
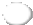
 Não

## 27. Você adota medidas de prevenção e controle da COVID-19 no seu convívio familiar?


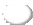

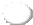
 Sim
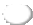
 Não

Não se aplica

## 28. Quais medidas você adota para prevenir a COVID-19 no seu convívio familiar (assinale mais de uma opção)?

Uso de máscara de tecido Uso de máscara N95 Higiene das mãos

Limpeza do ambiente do domicílio Distanciamento físico dos familiares Separação dos utensílios domésticos Isolamento domiciliar

Mudança de domicílio Higiene dos alimentos Não se aplica Nenhuma

Outra

## 29. Você teve diagnóstico de COVID? (clínico ou laboratorial)


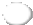
 Sim
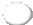
 Não

## 30. Na pandemia ficou isolado por um período da família?


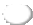
 Sim
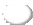
 Não


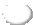
 Não se aplica

## 31. Você recebeu alguma capacitação ou curso sobre o COVID-19?


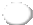
 Sim
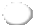
 Não

## 32. A instituição que você trabalha forneceu EPI suficiente para o uso?


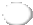

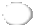
 Sim
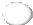
 Não

Em parte

## 33. A instituição que você trabalha forneceu EPI de boa qualidade?


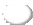
 Sim
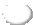
 Não


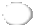
 Em parte

## 34. Quais EPI você usa na assistência às pessoas com suspeita ou diagnóstico de COVID-19?

Máscara cirúrgica Avental impermeável Avental de tecido Máscara N95

Luvas

Óculos de proteção

Protetor facial ou face shields Gorro

Pró pé Macacão Não se aplica

## 35. Em caso de procedimentos que geram aerossóis, quais EPI você usa em situação de COVID-19?

Máscara cirúrgica Avental impermeável Avental de tecido Máscara N95

Luvas

Óculos de proteção

Protetor facial ou face shields Gorro

Pró pé Macacão Não se aplica

## 36. Com que frequência você troca a máscara N95?


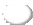
 A cada atendimento
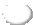
 Não se aplica


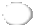
 Outra

##
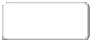
37. Caso tenha respondido a opção "outra" indique a cada quantos dias você troca a máscara N95:

- - 38. Qual o principal motivo que te leva a trocar a máscara N95?


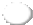
 Umidade


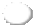
 Perda de vedação
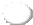
 Tempo de uso


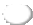
 Contaminação


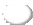
 Danificação (rasgo)
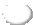
 Rotina estabelecida
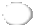
 Outra


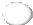
 Não se aplica

## 39. Com que frequência você troca a máscara cirúrgica na assistência a paciente com COVID-19?


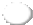
 No término do plantão
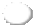
 De hora em hora


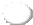
 De 2 em 2 horas


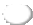
 De 3 em 3 horas


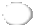

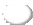
 De 4 em 4 horas Outra frequência

Não se aplica

## 40. Qual sequência que você adota durante a paramentação dos EPI no cuidado ao paciente com suspeita ou diagnóstico da COVID-19?

Avental, gorro, máscara, óculos de proteção ou protetor facial e luvas Máscara, óculos de proteção ou protetor facial, luvas, avental e gorro Óculos de proteção ou protetor facial, máscara, luvas, avental e gorro Gorro, máscara, avental, luvas, óculos de proteção ou protetor facial Não sigo uma sequência para a paramentação dos EPI

Não sei

Não se aplica Outro

## 41. Qual é a sequência da desparamentação após cuidado ao paciente com suspeita ou diagnóstico da COVID-19?

Luvas, máscara, avental, óculos de proteção ou protetor facial, e gorro Luvas, máscara, óculos de proteção ou protetor facial, avental e gorro Luvas, avental, óculos de proteção ou protetor facial, máscara e gorro

Avental, luvas, óculos de proteção facial ou protetor facial, gorro e máscara Não sigo uma sequência para a desparamentação dos EPI

Não sei

Não se aplica

## 42. Você teve alguma alteração na pele relacionada ao uso de máscara N95?

Sim Não

Não se aplica

## Qual foi o tipo?

Prurido Hiperemia Ressecamento Flictema/bolha Lesão

Não se aplica

## Qual o local?

Bochecha Nariz Orelha Queixo Nenhum

## 45. Teve alguma alteração na pele relacionada ao uso de óculos de proteção/face shield?

Sim Não

Não se aplica

## Caso sim, qual o local?

Bochecha Nariz Orelha Testa Nenhum

## Qual tipo?

Prurido Hiperemia Ressecamento Flictema/bolha Lesão Nenhum

## 48. Qual tipo de avental você usa na assistência dos pacientes com COVID-19?

Tecido com mangas longas Tecido com mangas curtas Impermeável com mangas longas Impermeável com mangas curtas Plástico com mangas longas Plástico com mangas curtas Nenhum

# Marque a resposta que corresponde com o que você sente ou tem sentido.

**Existem quatro opções de respostas: nem um pouco; não mais que o habitual; mais que o habitual e muito mais que o habitual**

## 49. Nos últimos três meses, pense especificamente na Pandemia do COVID

### nem um pouco não mais que o habitual mais que o habitual

**muito mais que o habitual**

Tem sido capaz de concentrar-se bem no que faz?

Tem perdido o sono por preocupações?

Tem se sentido útil para os demais?

Tem se sentido capaz de tomar decisões?

Tem se sentido constantemente sob tensão?

Tem sentido que não

pode solucionar seus problemas?

Tem sido capaz de apreciar a vida diária?

Tem sido capaz de

enfrentar seus problemas?

Tem se sentido triste ou deprimido?

Tem perdido confiança em si mesmo?

Tem sentido que você não vale nada?

Tem se sentido feliz

considerando todas as coisas?

## 50. Nos últimos três meses, alterou algum aspecto da sua vida decorrente da Pandemia?

### Melhorou Piorou Sem alterações

Na vida afetiva

No relacionamento com os amigos

No relacionamento com parceiros

Nas tarefas cotidianas de casa

Na vida sexual

Na satisfação sexual

No cuidado com sua saúde

Na alimentação

Alteração no peso corporal

Qualidade de vida

## 51. Para responder as próximas questões, considere os últimos três meses.

### Nunca Raramente Ocasionalmente Frequentemente Sempre

Você tem tido uma sensação forte e constante de falta de energia?

Você tem observado que precisa de mais energia para dar conta das suas tarefas diárias?

Você tem se sentido sem disposição para fazer as coisas?

Você tem acordado com

a sensação de estar exausto e desgastado?

Você tem tido necessidade de descansar mais?

Você tem conseguido

fazer suas atividades do dia-a-dia?

Seu interesse por sexo, sua vontade de ter relações sexuais diminuiu?

Tem sido mais difícil se

concentrar em uma coisa por muito tempo?

## 52. Para cada afirmação você poderá responder uma das opções: Quase nunca; Ocasionalmente; Muitas vezes; Com muita frequência e quase sempre.

### Quase nunca Ocasionalmente Muitas vezes

**Com muita**

**frequência Quase sempre**

Procuro formas criativas de superar situações difíceis

Independentemente do que me possa acontecer

acredito que posso

controlar as minhas reações

Acredito que posso crescer positivamente lidando com situações difíceis

Procuro ativamente formas de substituir as perdas que encontro na vida

# Você está quase terminando.

## 53. Na sua vida qual(is) dessa(s) substâncias você já usou (somente uso não prescrito pelo médico)

### Sim Não

Derivados do tabaco

Bebidas alcoólicas

Hipnóticos/sedativos

Outras

## 54. Durante os três últimos meses, com que frequência você utilizou alguma dessas substâncias?

### Diariamente ou quase todos os

**Nunca 1 ou 2 vezes Mensalmente Semanalmente dias**

Derivados do tabaco

Bebidas alcoólicas

Hipnóticos/sedativos

Outras

## 55. Nesse período de pandemia o uso dessa(s) substâncias aumentou? (somente uso não prescrito pelo médico)

### Sim Não

Derivados do tabaco

Bebidas alcoólicas

Hipnóticos/sedativos

Outras

Não se aplica

***Você quase terminou. Falta muito pouco. Conclua, por favor.***

## 56. Considerando o período da pandemia responda as questões abaixo pensando na sua prática clínica atual. Para cada afirmação você poderá assinalar uma das seguintes opções: Nunca; Raramente; Às vezes; sempre.

### Nunca Raramente Às vezes Sempre

Eu lavo minhas mãos entre contatos com pacientes

Eu uso somente água para lavar as mãos

Eu uso produto à base de álcool para higienizar as mãos como alternativa se não estiverem visivelmente sujas

Eu reencapo agulhas

usadas após aplicar uma injeção

Eu descarto materiais perfurocortantes em caixas próprias

A caixa de materiais perfurocortantes é descartada somente quando está cheia

Eu retiro os Equipamentos de Proteção Individual (EPI) em um local designado

Eu tomo banho em caso de respingos extensos mesmo que eu tenha usado EPI

Eu cubro meus ferimentos ou lesões com curativos a prova d

´agua antes do contato com pacientes

Eu uso luvas quando estou exposto a fluidos

corporais, sangue ou

derivados e qualquer excreção de pacientes

Eu troco de luvas entre contato com pacientes

### Nunca Raramente Às vezes Sempre

Eu higienizo as minhas

mãos imediatamente após remover as luvas

Eu uso máscara cirúrgica ou em combinação com óculos de proteção e avental sempre que houver a possibilidade de respingos ou derramamentos

Minha boca e meu nariz

ficam cobertos quando uso máscara

Eu reutilizo uma máscara cirúrgica ou EPI descartável

Eu uso avental/capote quando estou exposto a

sangue, fluídos

corporais ou qualquer excreção de pacientes

Eu descarto material contaminado com sangue, fluídos corporais, secreções e excreções de pacientes em sacos plásticos brancos

independente do estado infeccioso do paciente

Eu descontamino superfícies e

equipamentos após o uso

Eu uso luvas para descontaminar equipamentos que apresentam sujeira visível

Eu limpo imediatamente com desinfetante (álcool) superfícies após derramamento de sangue ou outros fluídos corporais

## 57. Considerando o período da pandemia responda as questões abaixo.

### Mais ou menos

**Nada eficaz eficaz Totalmente eficaz**

As precauções-padrão são medidas eficazes para reduzir as infecções hospitalares

Se eu seguir as precauções-padrão,

protegerei meus

pacientes de uma infecção

A maioria dos meus colegas de trabalho pensa que é importante seguir às precauções- padrão

Corro o risco de receber advertências dos meus

superiores, se não

seguir às precauções- padrão

Corro o risco de receber advertências dos enfermeiros e auxiliares responsáveis pela higienização, se não seguir às precauções- padrão

Corro o risco de receber advertências dos médicos, se não seguir às precauções-padrão

## 58. Que fatores (ou circunstâncias) facilitam a aplicação das precauções-padrão?

### Mais ou menos

**Nada eficaz eficaz Totalmente eficaz**

Ter material (qualidade, disponibilidade e acessibilidade) em todos os locais de trabalho

Estar capacitado no que

se refere às precauções- padrão

Ter capacitação quanto às precauções-padrão

Quando o profissional médico tem um

comportamento

exemplar em relação às precauções-padrão

Quando meus colegas de trabalho têm um comportamento exemplar em relação às precauções-padrão

## 59. Que fatores (ou circunstâncias) dificultam a aplicação das precauções-padrão?

### Mais ou menos um

**Não é obstáculo obstáculo É um obstáculo**

Situações inesperadas que podem atrapalhar a realização de meu trabalho (urgência, solicitação de colegas, nova tarefa a cumprir)

Falta de tempo

Carga de trabalho mais elevada que de costume

Complexidade das

medidas de precauções- padrão

Falta de conhecimento sobre as precauções- padrão

Rotina, hábitos e equipe de trabalho

Crenças pessoais relacionadas

às precauções-padrão

Problemas relacionados ao material (qualidade, disponibilidade e acessibilidade)

## 60. Tenho a intenção de seguir o protocolo das precauções-padrão do hospital:

### Nunca Raramente Às vezes Muitas vezes Sempre

Mesmo quando o paciente for difícil

Mesmo quando houver pouco tempo

Mesmo quando minhas mãos estiverem doloridas ou machucadas

Mesmo em uma situação de urgência
